# Enhancer of zeste homolog 2 silencing inhibits tumor growth and lung metastasis in osteosarcoma

**DOI:** 10.1038/srep12999

**Published:** 2015-08-12

**Authors:** Yang-Fan Lv, Guang-Ning Yan, Gang Meng, Xi Zhang, Qiao-Nan Guo

**Affiliations:** 1 Department of Pathology, Xinqiao Hospital, Third Military Medical University, Chongqing 400037, People’s Republic of China

## Abstract

The enhancer of zeste homolog 2 (*EZH2*) methyltransferase is the catalytic subunit of polycomb repressive complex 2 (PRC2), which acts as a transcription repressor via the trimethylation of lysine 27 of histone 3 (H3K27me3). *EZH2* has been recognised as an oncogene in several types of tumors; however, its role in osteosarcoma has not been fully elucidated. Herein, we show that EZH2 silencing inhibits tumor growth and lung metastasis in osteosarcoma by facilitating re-expression of the imprinting gene tumor-suppressing STF cDNA 3 (*TSSC3*). Our previous study showed that *TSSC3* acts as a tumor suppressor in osteosarcoma. In this study, we found that EZH2 was abnormally elevated in osteosarcoma, and its overexpression was associated with poor prognosis in osteosarcoma. Silencing of EZH2 resulted in tumor growth inhibition, apoptosis and chemosensitivity enhancement. Moreover, suppression of EZH2 markedly inhibited tumor growth and lung metastasis *in vivo*. Furthermore, EZH2 knockdown facilitated the re-expression of *TSSC3* by reducing H3K27me3 in the promoter region. Cotransfection with siEZH2 and siTSSC3 could partially reverse the ability of siEZH2 alone. We have demonstrated that EZH2 plays a crucial role in tumor growth and distant metastasis in osteosarcoma; its oncogenic role is related to its regulation of the expression of *TSSC3*.

Osteosarcoma is the most common primary malignant bone tumor^1^, and is the second highest cause of cancer-related death in children and young adolescents^2^,^3^,^4^. In recent years, the emergence of neoadjuvant chemotherapy dramatically increased the five-year survival rate. However, osteosarcoma remains a malignancy with a very high case fatality rate and disability rate. The progress of osteosarcoma treatment has been painfully slow for the past 20 years. Therefore, developing more targeted treatment is imperative.

The enhancer of zeste homolog 2 (*EZH2*) methyltransferase is the catalytic subunit of polycomb repressive complex 2 (PRC2), acting as a transcription repressor via trimethylation of lysine 27 of histone 3 (H3K27me3), often at the sites of tumor suppressor genes^5^,^6^. This epigenetic modification leads to dense packaging of chromatin making it less accessible to the transcriptional machinery, eventually resulting in silencing of these genes^7^,^8^. Therefore, *EZH2*-mediated methylation may be a potential independent mechanism for epigenetic silencing of tumor suppressor genes in cancer^9^. Actually, *EZH2* has been recognised as an oncogene in several types of tumors, such as breast cancer, prostate cancer, bladder cancer and oral squamous cell carcinoma, which makes EZH2 a potential biomarker in tumors^10^,^11^,^12^,^13^. Knockdown of EZH2 substantial reduces tumor growth and cell proliferation in multiple cancers^12^,^14^,^15^. Furthermore, pharmacologic interference of EZH2 function induces selective apoptosis in cancer cells but not in normal cells, making it an attractive anti-cancer drug target. It has been reported that EZH2 may be associated with distant metastasis and poor prognosis of human osteosarcoma^16^,^17^; however, previous work has demonstrated that the knock-down of overexpressed EZH2 did not prevent osteosarcoma growth^18^, suggesting that EZH2 might not be a useful molecular target for osteosarcoma treatment. Therefore, the association between EZH2 and human osteosarcoma still warrants further investigation.

In our previous study, we found that tumor-suppressing STF cDNA 3 (*TSSC3*) acts as a tumor suppressor in human osteosarcoma^19^,^20^. *TSSC3* is an imprinting gene, the expression of which is mainly regulated by epigenetic mechanisms, which aroused our attention. Our follow-up studies^21^ showed that hypermethylation of the *TSSC3* promoter region led to loss of expression in osteosarcoma cells. However, other related epigenetic regulatory mechanisms of *TSSC3* needs to be further characterised. The association between *EZH2* and *TSSC3* expression has not been investigated in osteosarcoma. We postulated that there might be a correlation between *EZH2* and the expression *TSSC3*. In this study, we demonstrate for the first time in osteosarcoma that EZH2 knockdown inhibits cell proliferation, migration and invasion *in vitro*, and growth of tumor xenografts and lung metastasis *in vivo* by facilitating the re-expression of *TSSC3*, activating the corresponding apoptosis pathway. Thus, these results provide a novel molecular mechanism for tumor growth inhibition.

## Results

### EZH2 is overexpressed in human osteosarcoma tissues and cell lines

**Figure 1:**
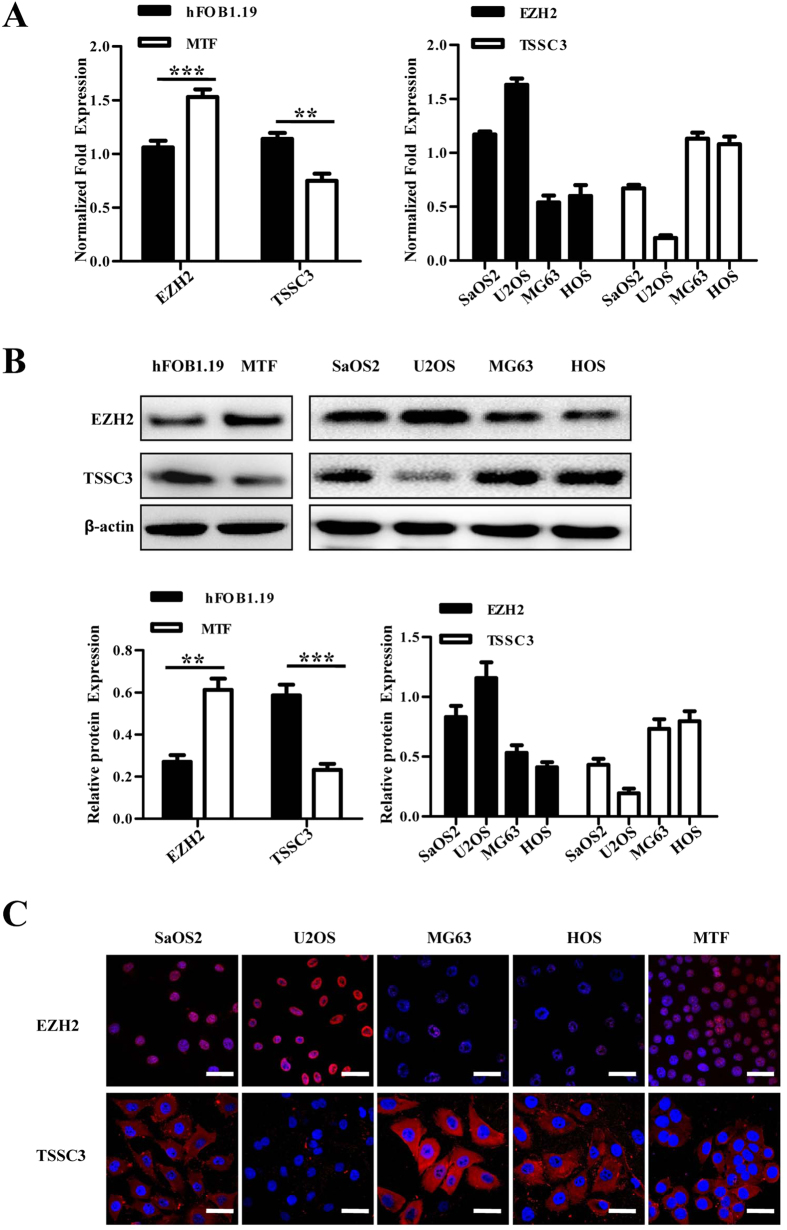
EZH2 is overexpressed in human osteosarcoma cell lines. (**A**) EZH2 mRNA expression was determined in hFOB1.19 osteoblasts, MTF osteoblasts and osteosarcoma cell lines (SaOS2, U2OS, MG63 and HOS) by qRT-PCR analysis. EZH2 expression was significantly increased in MTF cells compared with hFOB1.19 osteoblasts, whereas TSSC3 expression was notably decreased. Values shown are the mean ± SD from three wells from a representative of three independent experiments. ***P < 0.001; **P < 0.01; bars, SD. (**B**) Western blot analysis showing EZH2 and TSSC3 expression in hFOB1.19, MTF and osteosarcoma cells (upper). The results were consistent with that of qRT-PCR. Blots are representative of three experiments and were reprobed for β-actin to verify equal loading (lower). Band intensities were measured by densitometry and normalised to β-actin expression. (**C**) Osteosarcoma cell lines were immunostained with goat anti-EZH2 (red) and rabbit anti-TSSC3 antibody (red) and Hoechst33258 (blue) for observation by laser confocal microscopy. Scale bars: 50 μm.

The expression of *EZH2* in MTF cells was substantially higher than hFOB1.19 osteoblasts (Fig. 1A,B), suggesting that *EZH2* was upregulated during osteoblast malignant transformation. Furthermore, *EZH2* expression was contrary to the expression of *TSSC3* as previously described^19^.

Previous studies have reported that the expression of EZH2 is associated with tumor progression in multiple types of cancer^22^,^23^. To confirm this in osteosarcoma, real-time quantitative RT-PCR (qRT-PCR), immunofluorescence and western blot analysis were applied. The results showed that the expression of EZH2 appeared to be much higher in the cell lines derived from high-grade osteosarcoma (MTF, U2OS and SaOS2), meanwhile it was much lower in cell lines derived from low-grade osteosarcoma (MG63 and HOS; Fig. 1A–C).

**Figure 2:**
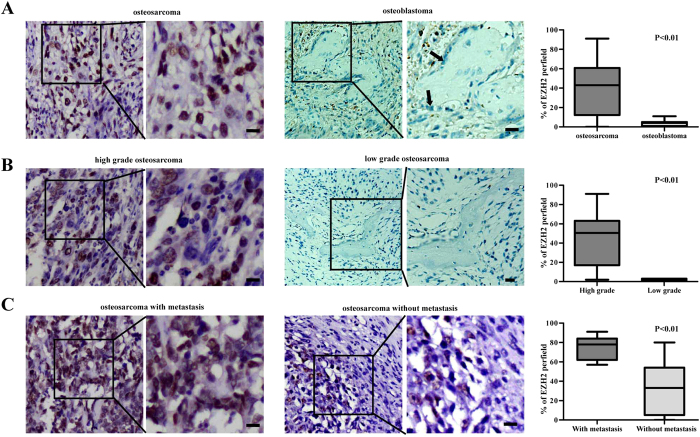
Immunohistochemistry staining of EZH2 in human osteosarcoma tissues. (**A**) Representative images of EZH2 expression (200×) in osteosarcoma and osteoblastoma. EZH2 expression is notably higher in osteosarcoma than osteoblastoma. In osteoblastoma, although some of the inflammatory cells and surrounding red cells showed nuclear staining of EZH2, the osteoblasts are absent of EZH2 expression (indicated by arrows). Quantitative comparison of EZH2 expression between osteosarcoma and osteoblastoma, p < 0.01 (right). (**B**) Representative images of EZH2 expression in high-grade and low-grade osteosarcoma. EZH2 expression is higher in high-grade osteosarcoma than low-grade. Quantitative comparison of EZH2 expression between high-grade and low-grade osteosarcoma, p < 0.01 (right). (**C**) Representative images of EZH2 expression in osteosarcoma with or without metastasis. EZH2 is higher in osteosarcoma with metastasis than without metastasis. Quantitative comparison of EZH2 expression between osteosarcoma with or without metastasis, p < 0.01 (right). Scale bars: 50 μm.

To determine the expression of EZH2 in osteosarcoma, immunohistochemistry (IHC) was performed in the tumor samples of osteosarcoma tissues. Based on the criteria, samples containing more than 50% of EZH2 or TSSC3 positive cells were designated as “positive expression”^24^,^25^. The EZH2 positive expression rate in osteosarcoma was 48.75%, notably higher than the osteoblasts in osteoblastoma, which is from the benign lesions in bone (P < 0.01). Figure 2 shows representative images of EZH2 osteosarcoma and osteoblastoma tissues. Strong nuclear EZH2 staining was seen in osteosarcoma and absence of EZH2 staining was found in osteoblastoma (Fig. 2A). In addition, the results revealed that EZH2 expression was higher in high-grade osteosarcoma than low-grade, and more evident in tissues with metastasis than without metastasis (Fig. 2B,C). Further statistical results revealed that EZH2 expression was negatively associated with that of TSSC3 (supplementary Table 1). Supplementary Figure S2 shows representative images of positive and negative TSSC3 expression (200×) in osteosarcoma.

### Association between EZH2 expression and the clinicopathologic features of osteosarcoma patients

EZH2 expression is significantly associated with the Enneking stage of osteosarcoma (P < 0.01, Pearson χ^2^ test; Table 1), grade and lung metastasis, whereas the results related to sex, age, tumor size, local recurrence and pathological subtypes are negative (Table 1). The Enneking system is based on the histological grade of the tumor, its local extent and the presence or absence of metastasis^26^. Only parosteal osteosarcoma and well-differentiated intramedullary osteosarcoma are defined as “low-grade”. Positive expression of EZH2 was frequently observed in high-grade osteosarcoma (52.7%), but not in low-grade osteosarcoma (0%). Moreover, 100% of stage III osteosarcoma patients showed positive expression of EZH2, whereas no positive result was detected in stage I tumors. Furthermore, EZH2 was highly expressed in samples with lung metastasis (100% over 38.81%), suggesting EZH2 might be involved in the distant metastasis process of osteosarcoma. Supplementary Figure S3 shows quantitative comparison of EZH2 expression in osteosarcoma tissues based on clinicopathologic features.

### EZH2 knockdown inhibits cell growth *in vitro* and *in vivo*

**Figure 3:**
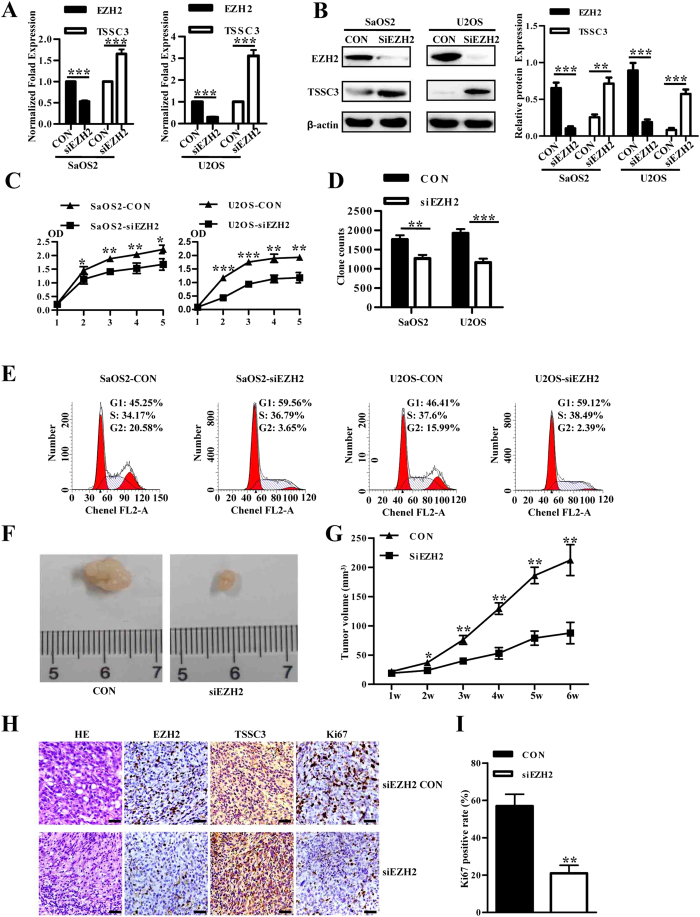
EZH2 knockdown inhibits cell growth of osteosarcoma *in vitro* and *in vivo*. (**A**) The expression of EZH2 and TSSC3 in SaOS2 and U2OS cell lines transfected with lenti-shEZH2 was evaluated by qRT-PCR. (**B**) Decreased protein level of EZH2 and increased TSSC3 was revealed by western blotting after transfection with lenti-shEZH2, consist with that of qRT-PCR (left). Blots are representative of three experiments and were reprobed for β-actin to verify equal loading (right). Band intensities were measured by densitometry and normalised to β-actin expression. (**C**) Downregulation of EZH2 caused a significant growth inhibition of osteosarcoma cells as revealed by CCK-8 assay. Lenti-siEZH2 versus control group, P < 0.05. Values shown are the mean absorbance ± SD for five wells from one experiment, and are representations of three independent experiments. (**D**) Inhibition of colony-formation by EZH2 knockdown in osteoblast cells. Values shown are mean ± SD from three independent experiments. ***P < 0.001 bars, SD. (**E**) DNA content analysis revealed that EZH2 knockdown increased the percentage of cells in G1 phase and decreased the percentage of cells in G2 phase in both SaOS2 (upper) and U2OS (lower) cell lines. The figure shows relative cell percentage in each cell cycle phase. Values shown are the mean ± SD from three independent experiments. ***P < 0.001; **P < 0.01; *P < 0.05; bars, SD. (**F**) Xenograft studies using lenti-shEZH2 transfected cells shows a reduced ability to form tumors in SCID mice. (**G**) Knockdown of EZH2 inhibited tumor growth of osteosarcoma *in vivo* (P < 0.05). (**H**) Hematoxylin-eosin and immunohistochemical staining with antibodies to EZH2, TSSC3 and Ki67 were performed on paraffin-embedded tumor sections of xenografts from siEZH2 cells and control cells. Scale bars: 100 μm. (**I**) Quantitation of Ki67 staining in xenografts from siEZH2 cells and control cells. **P < 0.01. Values are means ± SD from three independent experiments. Bars, SD.

Since the overexpression of EZH2 is associated with a more aggressive osteosarcoma tumor phenotype, we performed a CCK-8 assay, soft agar assay and cell cycle assay to determine whether EZH2 silencing can inhibit cell growth, colony formation and cell cycle arrest of osteosarcoma cells. SaOS2 and U2OS cell lines were transfected with lenti-siEZH2, and both the qRT-PCR and western blot showed EZH2 expression was efficiently reduced by siEZH2, whereas TSSC3 expression was increased (Fig. 3A,B). The CCK-8 assay showed that EZH2 knockdown cells grew more slowly than control, and the difference was more notable after day 2 or 3 when essentially all cells had established cell-cell contacts (Fig. 3C). The results of the soft agar assay showed a similar result (Fig. 3D). These results indicate a positive correlation between the expression of EZH2 and osteosarcoma cell growth. DNA content detected by flow cytometry demonstrated that EZH2 knockdown increased the cell percentage in G1 phase and decreased those in G2 phase in SaOS2 and U2OS cells (Fig. 3E).

**Figure 4:**
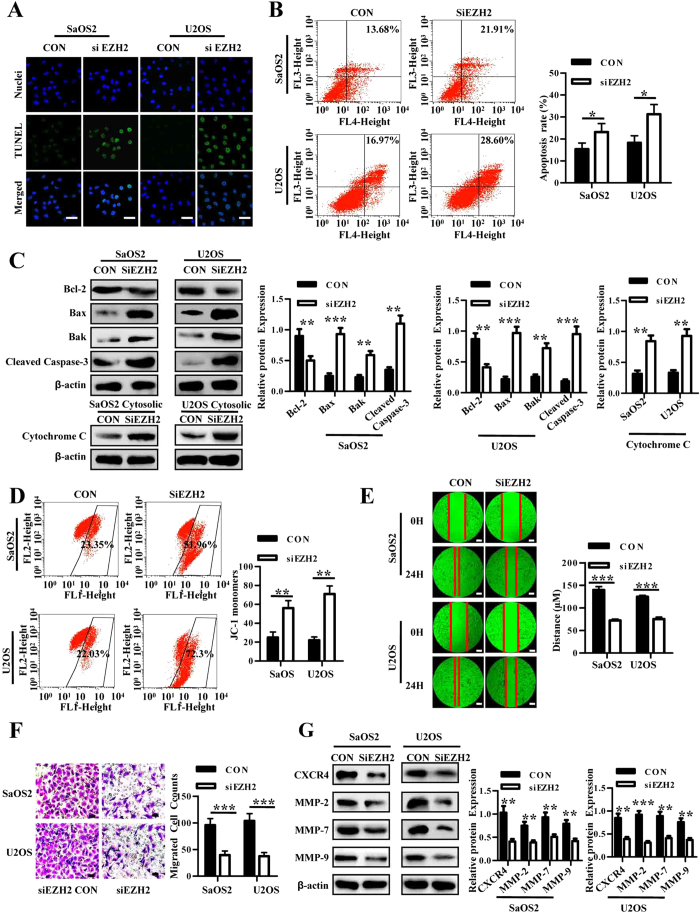
EZH2 silencing induces apoptosis and inhibits migration and invasion in osteosarcoma. (**A**) Lenti-shEZH2 and control vector transfected SaOS2 and U2OS cells were immunostained with TUNEL antibody (green) and Hoechst33258 (blue); scale bars 50 μm. (**B**) Lenti-shEZH2 and control vector transfected cells were stained with Annexin V-APC and 7-AAD for flow cytometric analysis. Shown are representative images of three independent experiments. Bar graph (right) of flow cytometric analysis showing mean ± SD from three independent experiments is shown. *P < 0.05. (**C**) Western blot analysis of Bcl-2, Bax, Bak and cleaved caspase 3 proteins in SaOS2 and U2OS cells with or without EZH2 knockdown. Blots are representative of three experiments and were reprobed for β-actin to verify equal loading (right). Band intensities were measured by densitometry and normalised to β-actin expression. (**D**) Mitochondrial transmembrane potential assay. Osteosarcoma cells with or without EZH2 silencing were cultured under suspension and then stained with JC-1 solution for flow cytometric analysis. Shown are representative images of three independent experiments. Bar graph (right) of flow cytometric analysis showing mean ± SD from three independent experiments is shown. **P < 0.01. (**E**) Cell migration by wound healing assay in SaOS2 and U2OS cells. The photographs were taken at 0 h and 24 h, respectively. Y axis of bar graph of wound healing (right) is migration distance of tumor cells in 24 h. ***P < 0.001 bars, SD. Scale bars: 50 μm. (**F**) Trans-well migration assay using lenti-shEZH2 and control vector transfected SaOS2 and U2OS cells. The migrated cells were stained with crystal violet and counted. Bar graph of trans-well migration assay representing the mean value ± SD from independent experiments performed in triplicate. ***P < 0.001. Scale bars: 50 μm. (**G**) Western blot analysis of CXCR4, MMP-2, MMP-7 and MMP-9 proteins in osteosarcoma cells with or without EZH2 knockdown (left). Blots are representative of three experiments and were reprobed for β-actin to verify equal loading (right). Band intensities were measured by densitometry and normalised to β-actin expression.

Tumor formation was detected in all 6 nude mice inoculated with SaOS2 control cells and lenti-siEZH2 cells. However, tumor xenografts formed from the latter were distinctly smaller than control tumors throughout the study period (Fig. 3F,G). Hematoxylin-eosin stained specimens obtained from subcutaneous tumors are shown in Fig. 4C. The SaOS2 control cell-derived tumors contained numerous nuclei with karyokinesis, pleomorphism and high density of microvessels (Fig. 3H), typically seen in human osteosarcoma. In tumors formed by siEZH2 cells, less karyokinesis and low microvessel densities were observed. As expected, tumors derived from the siEZH2 cells demonstrated lower EZH2 expression and higher TSSC3 expression (Fig. 3H). Furthermore, the frequency and intensity of Ki67 expression in tumors derived from siEZH2 cells were significantly lower than the control group (59% over 21%; P = 0.001, Student’s t-test; Fig. 3H,I). Altogether, these results suggested a role of EZH2 silencing in tumor growth inhibition *in vivo*.

### EZH2 knockdown induces apoptosis, inhibits migration and invasion in osteosarcoma

To determine whether the reduction in growth of EZH2 knockdown cells might be due to an increase in apoptosis, TUNEL assay and annexin V/7-AAD analysis were performed. The TUNEL assay showed that the frequency and intensity of FITC-positive cells in siEZH2 cells was substantially higher than in control cells (Fig. 4A). Additionally, the percentage of late apoptotic cells prominently increased in cells with EZH2 silencing (Fig. 4B). By western blot, we observed a decrease in the anti-apoptotic Bcl-2, and an increase in the pro-apoptotic Bax and Bak proteins, with an increased level of cleaved caspase 3 in cells transfected with the siEZH2 lentivirus (Fig. 4C). Furthermore, knockdown of EZH2 caused an increase in the expression of cytochrome C in the cytosol, indicting an enhancement of the intrinsic apoptotic pathway (Fig. 4C). Similar results were obtained from mitochondrial membrane potential analysis (Fig. 4D).

The wound healing results suggested that the silencing of EZH2 could severely inhibit migration of osteosarcoma cells (Fig. 4E). Meanwhile, silencing EZH2 in SaOS2 and U2OS cells showed reduced invasive potential compared with the control group (Fig. 4F). Lower levels of the osteosarcoma metastasis-associated proteins CXCR4, MMP-2, MMP-7 and MMP-9 were detected in siEZH2 cells (Fig. 4G).

### Inhibition of *in vivo* lung metastasis by siEZH2

**Figure 5:**
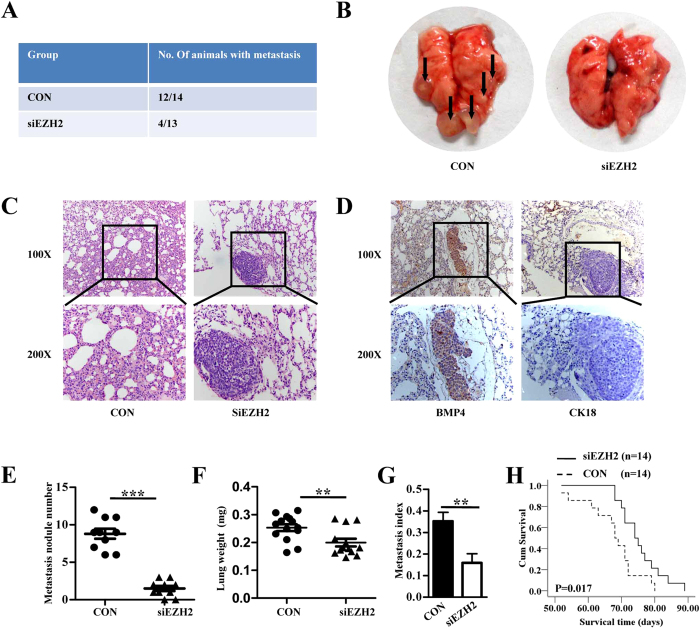
Inhibition of *in vivo* lung metastasis by EZH2 knockdown. (**A**) Number of mice bearing lung metastases. (**B**) Macroscopic appearances of lung metastasis are shown. Metastatic tumor nodules were identified as whitish and patchy areas. (**C**) Representative hematoxylin-eosin stained images of the lungs shown in B. (**D**) Representative IHC staining images of the lungs as shown in B. (**E**) Number of tumor nodules was evaluated and analysed between MTF cells transfected with lenti-shEZH2 and control group. ***P < 0.001. (**F**) Lung metastasis index (ratio of tumor area to the total tumor and lung area) was analysed between siEZH2 and the control group. **P < 0.01. (**G**) Mean weight of the lungs. **P < 0.01. All values in E-G are mean + SD of the 14 mice in siEZH2 CON group and 13 mice in siEZH2 group. (**H**) EZH2 silencing led to later death of mice seen by survival curve analysis. *P < 0.05, n = 14.

EZH2 knockdown considerably inhibited lung metastasis of osteosarcoma cells, as reflected by the incidence, number, size and weight of the metastatic nodules (Fig. 5A–G). As expected, the lung nodules were CK18-negative and positive for BMP4, indicating their mesenchymal origin (Fig. 5D). In addition, silencing of EZH2 also led to later death of mice (Fig. 5H).

### Enhanced sensitivity of osteosarcoma cells to chemotherapeutic agents by EZH2 silencing through growth inhibition and promotion of apoptosis

**Figure 6:**
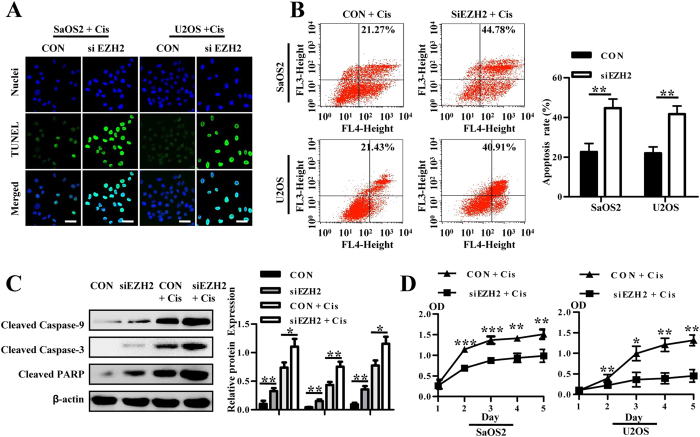
Silencing of EZH2 enhances the sensitivity of osteosarcoma cells to chemotherapeutic agents. (**A**) Lenti-shEZH2 and control vector transfected cells treated with cisplatin were immunostained with the TUNEL antibody (green) and Hoechst33258 (blue), scale bars 50 μm. (**B**) Lenti-shEZH2 transfected cells treated with cisplatin showed higher apoptosis rates than control group, which is representative of three independent experiments. Bar graph of apoptosis (right) rate is shown. (**C**) Western blot analysis of cleaved caspase 9, cleaved caspase 3, and cleaved PARP proteins in osteosarcoma cells (left). Blots are representative of three experiments and were reprobed for β-actin to verify equal loading (right). Band intensities were measured by densitometry and normalised to β-actin expression. (**D**) CCK-8 analysis showed that after treatment with cisplatin, EZH2-silenced SaOS2 and U2OS cells showed a decrease in proliferation.

siEZH2 cells were treated with cisplatin, followed by CCK-8, TUNEL and annexin V/7-AAD analysis. Silencing of EZH2 increased apoptosis in osteosarcoma cells treated with cisplatin, indicated by TUNEL assay and annexin V/7-AAD analysis (Fig. 6A,B). Western blot analysis confirmed the above result (Fig. 6C). Additionally, they grew more slowly than the control, whereas cell proliferation of both groups decreased substantially after cisplatin treatment (Fig. 6D). Altogether, these results suggest that knockdown of EZH2 increases the sensitivity of osteosarcoma cells to chemotherapeutic agents.

### EZH2 silencing enhances expression of *TSSC3*, and *TSSC3* is a direct target of H3K27me3

**Figure 7:**
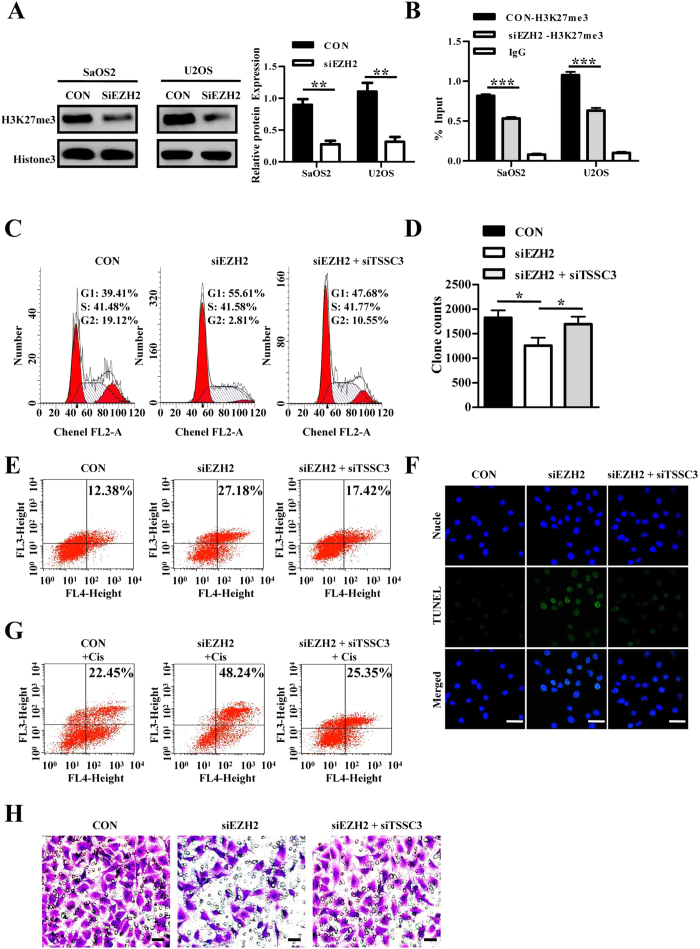
EZH2 repressed TSSC3 expression via H3K27me3 modification and inhibition of TSSC3 partially rescues the impaired biological function of EZH2 silencing. (**A**) Western blot analysis of HK27me3 after lenti-shEZH2 transfection using total histone 3 as a reference (left). Blots are representative of three experiments and were reprobed for histone 3 to verify equal loading (right). Band intensities were measured by densitometry and normalised to histone 3 expression. (**B**) ChIP analysis shows the H3K27me3 levels within the TSSC3 promoter, which is inhibited by EZH2 silencing. IgG was utilised as a negative control. (**C**) EZH2 silencing lead to a G1 phase cell cycle arrest, which was decreased by TSSC3 knockdown. (**D**) Soft agar assays. (**E**) Apoptosis assays. (**F**) TUNEL assays. (**G**) Apoptosis assays are applied to indicated cells treated with cisplatin. (**H**) Transwell matrigel invasion assay. Scale bars: 50 μm.

Both the qRT-PCR and western blot results indicated that EZH2 silencing led to reduction of H3K27me3 and re-expressed *TSSC3* (Figs 3A,B and 7A). ChIP assays using an H3K27me3 antibody revealed its interaction with the *TSSC3* promoter. Moreover, this interaction was significantly inhibited by EZH2 silencing (Fig. 7B), indicating that a possible epigenetic transcriptional re-activation of *TSSC3* expression was conducted by EZH2. Supplementary Figure S9 shows full-length agarose gels from the ChIP assay.

### Repression of TSSC3 is potentially involved in the oncogenic function of EZH2

To determine whether TSSC3 is involved in EZH2’s regulation of osteosarcoma cell proliferation, metastasis and apoptosis, we performed rescue experiments. SaOS2 cells were cotransfected with siEZH2 and siTSSC3, and it was shown to partially reverse the ability of siEZH2. The cell cycle analysis, soft agar assay, annexin V/7-AAD analysis and TUNEL assay indicated that the cotransfection could partially reverse the siEZH2-induced biological effect, including cell growth inhibition, apoptosis induction, invasion suppression and enhancement of sensitivity to chemotherapeutic agents in SaOS2 cells (Fig. 7C–H). These data indicate that EZH2 promotes osteosarcoma cell proliferation, invasion and inhibits apoptosis through the downregulation of TSSC3 expression.

## Discussion

The *EZH2* histone methyltransferase is located on chromosome 7q35 and encodes a polycomb group protein. Multiple studies have detected abnormally high expression of EZH2 in several epithelial cancers, linking its overexpression to malignant tumor progression^24^,^27^,^28^. Recently, EZH2 has been identified as one of the novel genes harbouring mutations in T-cell prolymphocytic leukemia^29^, and its expression might also be involved in soft tissue sarcomagenesis^30^. In the present study, we examined the expression of EZH2 in osteosarcoma samples by IHC. The results indicated that EZH2 expression was highly expressed, suggesting its oncogenic role in osteosarcoma. Further correlation analyses demonstrated that high expression of EZH2 in osteosarcoma was positively correlated with an ascending histopathological grade, advanced clinical stage and distant tumor metastasis, suggesting that abnormal upregulated of EZH2 expression in osteosarcoma may facilitate the increased malignant phenotype of osteosarcoma and be a potential molecular biomarker for tumor prognosis. Similar results were also observed in other human malignancies, such as oesophageal, breast, lung, colorectal, bladder, ovarian, prostate cancers and others^10^,^14^,^22^,^23^,^24^,^31^,^32^, in which overexpression of EZH2 was often observed in more aggressive tumor subgroups and has diagnostic value. Therefore, the examination of EZH2 expression by IHC could be used as an additional effective tool in identifying those osteosarcoma patients at increased risk of tumor progression and/or metastasis. These findings highlight a potentially novel role for EZH2 as an underlying biological mechanism in the progression and/or metastasis of human osteosarcoma.

EZH2 was reported to be involved in several key regulatory mechanisms within eukaryotic cells, such as regulation of cell differentiation and cell proliferation^14^,^15^. It is the catalytic subunit of PRC2 and located in the nucleus, however, recently EZH2 has been detected in the cytoplasm and implicated in migration and invasion of cancer cells^33^,^34^,^35^. Notably, EZH2 appears to be not only a potential tumor biomarker but may itself contribute to the deregulation of tumor cells similar to an oncogene. Knockdown of EZH2 expression by antisense constructs, RNA interference or pharmacological inhibitors have resulted in growth inhibition in several types of cancer cells^13^,^24^. In the present study, using two human osteosarcoma cell lines, inhibition of EZH2 expression led to cell cycle arrest in G1 phase, reduced cell growth/proliferation *in vitro* and *in vivo*. Recently, Wee’s^13^ and Zhang’s^36^ work demonstrated inhibition of EZH2 expression induced apoptosis in prostate cancer, however, Rao’s^24^ study indicated inhibition of EZH2 expression did not affect apoptosis of ovarian carcinoma. Our data was similar to the result of Wee *et al*.^13^ in osteosarcoma, suggesting EZH2 has different roles in various types of cancer. These data provide evidence that EZH2 is not only importantly involved in osteosarcoma proliferation but also involved in tumor cell apoptosis.

Metastasis is regarded as a difficult problem in the treatment of osteosarcoma^37^,^38^, which is a multi-step process and cellular invasion contributes to it^39^,^40^. Several studies have found a correlation between high EZH2 levels and tumor relapse, distant metastasis, invasiveness and angiogenesis^31^,^41^,^42^,^43^,^44^,^45^. In line with these observations, our current study revealed considerable inhibition of invasion and migration after transfection of lenti-shEZH2 in osteosarcoma cells. Furthermore, the *in vivo* experiments verify the above conclusion. Therefore, EZH2 is a molecule that is involved in cancer cells migration and/or invasion and thus, the abnormal expression of EZH2 may provide a selective advantage in osteosarcoma cells invasiveness and/or metastasis.

However, the downstream molecular events involving EZH2 and osteosarcoma growth and/or metastasis molecular mechanisms remain unclear. However, the present data indicate EZH2’s important role in the regulation of *TSSC3* expression, which is an apoptosis-related gene and involved in malignant transformation of the human osteoblast^21^. Our laboratory previously found that TSSC3 could act as a tumor suppressor by negative growth regulation and cell apoptosis induction in osteosarcoma^19^. Epigenetic regulation is vital for imprinting genes and epigenetic silencing by histone methylation has been shown to play an important role in human cancer progression. Initial investigations in osteosarcoma cells indicated that EZH2 suppresses TSSC3 expression both at the transcript and protein levels, through histone H3K27 trimethylation at the TSSC3 promoter. Our rescue experiments performed by cotransfection of siEZH2 and siTSSC3 also confirmed the involvement of EZH2 in the mechanism that mediates TSSC3 repression during cancer progression. In the present study, we demonstrate the role for EZH2 in osteosarcoma from the aspect of *TSSC3*′s epigenetic regulation. However, EZH2 could act as a gene regulator, especially for tumor suppressor genes, as well as have the potential to be a direct executant. Therefore, the biological function of EZH2 could be related to other tumor suppressor genes or EZH2′s own effect. Further studies to investigate the exact mechanism by which EZH2 regulates the apoptotic pathway in osteosarcoma needs further examination. TSSC3 [also known as pleckstrin homology-like domain family A member 2 (*PHLDA2*)] is a PH domain-only protein, thus, by analogy with other PH domain proteins, TSSC3 mostly modulates cell signaling, intracellular trafficking or processes that depend on phosphatidylinositol lipid second messengers^46^. TSSC3 is reported to activate PI3K/AKT signaling via binding PIPs^46^, and a recent study indicated PHLDA3 (a homolog of *TSSC3*), is a repressor of AKT by directly interfering with AKT binding to PIPs^47^. Hence, we questioned if EZH2 promotes oncogenesis by activating the PI3K/AKT pathway. Intriguingly, our data showed that EZH2 knockdown leads to a decrease in the expression of phosphorylated AKT, which is reversed by cotransfection with siTSSC3 (supplementary Figure S10). Thus, the PI3K/AKT pathway may be involved in EZH2′s ability to promote tumor progression and metastasis in osteosarcoma. A recent study showed that PI3K-AKT regulates the phosphorylation of EZH2 to an inactive formation^13^. Therefore, the PI3K/AKT pathway is of importance in both EZH2 regulation and biological function in cancer development and further in depth study is necessary. It has also been previously reported that changes in the levels of specific microRNAs or intronic RNAs can influence *EZH2* expression^48^,^49^,^50^. Along these lines, our group will carry out work in this field.

Lenti-shEZH2 was employed to effectively knockdown the expression of EZH2 in osteosarcoma cells. RNAi-based therapies are especially appealing as they can target tumor-specific elements with less toxicity than traditional pharmacological approaches^51^. Moreover, resistance to commonly used drugs in osteosarcoma treatment remains a significant impediment to efficacious osteosarcoma therapy. Our results suggest that knockdown of EZH2 made osteosarcoma cells more sensitive to the cytotoxic effects of cisplatin. In the future, the use of less-toxic RNAi therapy may allow the use of lower doses of chemotherapy with greater efficiency, decreasing the side effects of these chemotherapeutics with a better outcome. Moreover, a combination that includes TSSC3 overexpression may be an additional pathway conducive to EZH2 RNAi therapy. Moreover, therapy targeting epigenetic machinery is coming to the forefront in cancer treatment^52^. The related pharmacodynamic study showed that 3-Deazaneplanocin A (DZNep), a known EZH2 inhibitor, can induce apoptosis in cancer cells, including prostate cancer^36^, breast cancer^53^ and acute myeloid leukemia^54^. DZNep is a histone methyltransferase inhibitor, which may disrupt PRC2 and deplete EZH2 and the associated H3K27me3. Therefore, the usage of DZNep or other epidrugs holds the potential for epigenetic treatment of cancer.

In summary, a positive relationship was found between EZH2 and tumor growth and metastasis of osteosarcoma. Reduced expression of EZH2 could also notably increase tumor cell apoptosis and enhance sensitivity to cisplatin via decreasing H3K27me3 levels in the *TSSC3* promoter region. Therefore, our findings suggest that siEZH2 holds promise as a potential therapeutic modality for osteosarcoma and warrants further study for possible clinical application. This is the first study that identifies the role of EZH2 in the growth and metastasis of osteosarcoma *in vivo* and its relationship with TSSC3.

## Methods

### Cell lines and cell culture

Immortalised human osteoblast cells hFOB (hFOB1.19) were purchased from American Type Culture Collection (ATCC; Manassas, VA, USA). Cells were cultured in Dulbecco’s minimal essential medium/Ham’s F12 (Hyclone, Logan, UT) with 10% fetal bovine serum (FBS; Gibco, Grand Island, NY) at 33.5 °C in a 5% CO_2_ incubator. The malignant transformed hFOB cell line (MTh.FOB cells, MTF) was established by our group^55^. Other human osteosarcoma cell lines, including SaOS2, MG63 and U2OS, were obtained from ATCC and cultured in Dulbecco’s modified Eagle’s medium (DMEM; Hyclone) supplemented with 10% FBS at 37 °C in a 5% CO_2_ incubator. For drug treatment, cells were treated with cisplatin (Sigma) for 24 h at a final concentration of 3 μg/ml.

### Construction of lenti-siEZH2

*EZH2* shRNA vector pLKD.CMV.GFP.U6 and non-target control shRNA were obtained from Neuron Biotech (Shanghai, China). The siEZH2 vector sequence is 5′-GTGCCCTTGTGTGATAGCACAA-3′ and 5′-GGCACTTTCATTGAAGAACTAA-3′. The non-target control vector sequence is 5′-TTCTCCGAACGTGTCACGT-3′.

### Patients and tissue specimens

In this study, 80 patients with osteosarcoma underwent surgical biopsy with no prior chemotherapy or radiation therapy in Xinqiao or Southwest Hospital in Chongqing, China between 2001 and 2009. Meanwhile, 30 patients with osteoblastoma were collected as benign tumor samples^56^.

### IHC staining

IHC was carried out by using an IHC kit (Zsbio, Beijing, China). Sample sections were deparaffinised through a series of xylene baths, antigen retrieved by steam treatment in 10 mM citrate buffer, blocked with 3% hydrogen peroxide for 15 min at 37 °C, pre-incubated with blocking serum solution for 30 min at 37 °C and then incubated at 4 °C with the primary antibodies overnight. Subsequently, the secondary antibodies were applied and the nuclei were counterstained with hematoxylin. The slides were then examined from nonoverlapping cells using an Olympus BX50 light microscope. For semi-quantitative assessment of protein expression, the percentage of positive cells was calculated in randomly selected, more than five higher-power fields, including over 100 cells, and the mean was regarded as the labelling index of each lesion. According to previous work^24^,^25^, samples containing more than 50% of EZH2 or TSSC3 positive cells were designated as “positive expression”.

### Immunofluorescence

Cells were plated onto coverslips in DMEM with 10% FBS for 24 h, fixed with 4% paraformaldehyde, incubated in 0.3% Triton X-100-PBS for 15 min, followed by blocking with 5% goat serum. The cells were then incubated with primary antibodies against EZH2 at 4 °C overnight, followed by appropriate secondary antibodies (Cy3 Red rabbit anti-goat and Cy3 Red goat anti-rabbit; Boster, Wuhan, China). The nuclei were counterstained with Hoechst 33258 (Beyotime, Shanghai, China). The immunofluorescent signals were detected by laser confocal scanning microscopy (SP-5, Leica, Germany).

### Soft agar assay

SaOS2 and U2OS cells transfected with the lenti-shEZH2 were examined using a colony formation in soft agar culture assay. Low melting-point agar (Invitrogen, Carlsbad, CA) was prepared at a concentration of 1.2%. Then, the agar was mixed with 2xDMEM and 20% FBS and allocated quickly at 2 ml/60 mm dish, making the final concentration of agar at 0.6%. A second upper agar was prepared in a similar process with 0.35% agar, also containing 1.5 × 10^4^ cells/ml. A total of 3 ml of the second agar was layered on the top of the first layer, placed in a 37 °C with 5% CO_2_ incubator for 30 min, allowing it to solidify. The plates were cultured for 14 days, and the colonies were counted under an inverted phase contrast microscope. Only colonies containing over 100 cells were presented in the final analysis.

### qRT-PCR analysis

Total RNA from cultured cells was extracted using RNAiso (Takara, Dalian, China) according to the manufacturer’s protocol. qRT-PCR was carried out using the SYBR Premix Ex TaqTM II (TaKaRa). All reactions were performed in a C1000TM Thermal cycler (Bio-Rad CFX96TM Real-time System). The results from qRT-PCR were normalised using the threshold cycle of GAPDH. Targeted genes were amplified with primers listed below: *EZH2*, 5′-GAAAGCCGCCCACCTC-3′ and 5′-AAACATCGCCTACAGAAAAGC-3′; *TSSC3*, 5′-TCCAGCTATGGAAGAAGAAGC-3′ and 5′-GTGGTGACGATGGTGAAGTACA-3′; *GAPDH*, 5′-CTTTGGTATCGTGGAAGGACTC-3′ and 5′-GTAGAGGCAGGGATGATGTTCT-3′.

### Western blot analysis

Proteins were separated using 8–15% SDS Tris-glycine gels and transferred onto a phenylmethylsulfonyl fluoride membrane (Millipore, USA). Membranes were blocked with 5% fat-free milk and incubated with antibodies against β-actin, EZH2, cleaved caspase 3, cleaved caspase 9, cleaved PARP (Cell Signaling Technology, Beverly, MA), TSSC3, Cytochrome C, CXCR4 (Abcam, Cambridge, UK), MMP-2, MMP-7, MMP-9 (Santa Cruz Biotechnology, Santa Cruz, CA), Histone 3 or H3K27me3 (Millipore) overnight at 4 °C. Then an appropriate secondary antibody (anti-mouse or anti-rabbit IgG; Zhong-shan, China) was applied. Immunoreactivity was detected using an ECL Kit (Pierce). The results were analysed using Image LabTM Software (Bio-Rad Laboratories).

### Cell migration and invasion assays

A wound healing assay was performed to investigate cell migration of osteosarcoma. After serum starvation overnight, monolayer cells at 80% confluency were disrupted mechanically with a pipette tip to generate a wound ridge free of cells, and the extent of cell migration was measured by the ability to close the artificially created gap, which was documented under a light microscope after 24 hours. A matrigel-based invasion assay was conducted using the Millipore invasion chamber (Millipore).

### Cell cycle analysis, TUNEL assay, and mitochondrial membrane potential analysis

TUNEL assay was performed by using an *in situ* cell death detection kit (Roche Biochemicals, Mannheim, Germany) following the manufacturer’s instructions. Cell cycle analysis was performed by using a cell cycle kit (Beyotime Institute of Biotechnology, Haimen, China) according to the manufacturer’s instructions. Changes in mitochondrial transmembrane potential occurring during apoptosis induced by siEZH2 or/and siTSSC3 transfection were examined using a mitochondrial membrane potential assay kit (Beyotime Institute of Biotechnology, Haimen, China) with lipophilic cation 5,5′,6,6′-tetrachloro-1,1′,3,3′-tetraethylbenzimidazolcarbocyanine iodide (JC-1). Briefly, cells were cultured in suspension for 2 days harvested and incubated with 1 ml JC-1 working solution in a CO_2_ incubator for 20 min at 37 °C. The cells were then washed with JC-1 staining buffer twice and then analysed by flow cytometry (FACSCalibur; Becton Dickinson, Franklin lakes, NJ).

### Apoptosis assay

Apoptosis assay was performed with an Annexin V-APC antibody (eBioscience, Santiago, CA, UT) and 7-AAD antibody (KeyGEN, Nanjing, China). Harvested cells were washed three times in PBS and then resuspended in 300 μl binding buffer (eBioscience). Subsequently, a total of 10 μl of 7-AAD and 5 μl of Annexin V-APC were added and incubated for 15 min. Then the stained cells were analysed by flow cytometry (10,000 cells; FACSCalibur).

### Chromatin immunoprecipitation analysis (ChIP)

ChIP was performed according to the manufacturer’s instructions using an EZ ChIP kit (Millipore) and anti-H3K27me3 antibody (Millipore). The immunoprecipitated DNA was tested by qRT-PCR as described above. Primers used for ChIP are as follows: 5′-TGAGCCCCGTCATTGAGGCCA-3′ and 5′-TCTCCCACCTTCCC GCACCC-3′.

### Mice xenograft and mouse model of osteosarcoma metastasis

Animal maintenance and experimental procedures conformed to the Institutional Animal Care and Use Committee of the Southwest Hospital, TMMU according to the Guide for the Care and Use of Laboratory Animals. Osteosarcoma cells (SaOS2) transfected with lenti-shEZH2 or control vectors (4 × 10^5^ cells) were injected subcutaneously into 5-week-old female severe combined immunodeficient (SCID) mice [n = 6 for control (CON) and siEZH2 cells, respectively; Laboratory Animal Center, Southwest Hospital, TMMU]. Subcutaneous xenograft size was measured every week after implantation, and the mice were killed 7 weeks later. Each of the SCID mice was injected with human MTF cells (with or without EZH2 knockdown; 7 × 10^6^ cells) through the tail vein. At 9 weeks after injection, the lungs were obtained and fixed in formaldehyde for histopathology analysis (n = 12 for CON cells, n = 11 for siEZH2 cells) or observed until their death for the survival curve (n = 14 for CON and siEZH2 cells, respectively). Furthermore, for all the mice used in our study, when they became moribund, euthanasia was performed to minimize any pain and distress they experienced.

### Statistical analysis

Statistical analyses were performed using the SPSS software (version 16.0; SPSS Inc., Chicago, IL). All experiments were repeated at least three times to calculate the mean and standard deviation (SD). The Student’s t-test was conducted for normally distributed data. The correlation between EZH2 and TSSC3 was assessed by the nonparametric (the Spearman ρ) correlation test. The correlation between EZH2 expression and clinicopathological characteristics was assessed by the Pearson χ^2^ test. Two-tailed tests were used for all hypothesis tests in the present study. A P-value < 0.05 was regarded as statistically significant and marked with an asterisk.

### Ethics statement

The study was carried out in accordance with the approved guidelines. The experiments were approved by the Institutional Ethics Committee of Third Military Medical University (TMMU). The study complied with the Declaration of Helsinki. All samples were obtained with written informed consent from all participants for publication of this study and any accompanying images.

## Additional Information

**How to cite this article**: Lv, Y.-F. *et al*. Enhancer of zeste homolog 2 silencing inhibits tumor growth and lung metastasis in osteosarcoma. *Sci. Rep*. **5**, 12999; doi: 10.1038/srep12999 (2015).

## Supplementary Material

Supplementary Information

## Figures and Tables

**Table 1:** Association between EZH2 expression and the clinic-pathological characteristics of osteosarcoma patients.

Characteristics	EZH2 protein		
	positive	Negative	Chi-square P-value
**Age (years)**			0.486
11~20	14	20	
21~30	13	10	
≥31	12	11	
**Gender**			0.385
Male	24	29	
Female	15	12	
**Grade**			0.039*
Low	0	6	
High	39	35	
**Stage**			<0.01*
I	0	6	
II	26	35	
III	13	0	
**Local recurrence**			0.334
Yes	9	6	
No	30	35	
**Lung metastasis**			<0.01*
Yes	13	0	
No	26	41	
**Histological type**			0.173
Osteoblastic	11	17	
Chondroblastic	14	18	
Fibroblastic	3	1	
Others	11	5	
**tumor size**			0.334
<10 cm	30	35	
≥10 cm	9	6	
