# Supplementary material for: Enhancer of zeste homolog 2 silencing inhibits tumor growth and lung metastasis in osteosarcoma
Source: Sci Rep. 2015 Aug 12;5:12999. doi: 10.1038/srep12999 (PMC4533017; doi:10.1038/srep12999)
Supplement: Supplementary Information [file srep12999-s1.pdf]

Supplementary information

Enhancer of zeste homolog 2 silencing inhibits tumor growth and lung metastasis in osteosarcoma

Yang-Fan Lv, Guang-Ning Yan, Gang Meng, Xi Zhang, Qiao-Nan Guo*

Department of Pathology, Xinqiao Hospital, The Third Military Medical University, Chongqing 400037, People's Republic of China

Supplementary Figures S1-S10
Supplementary Table 1

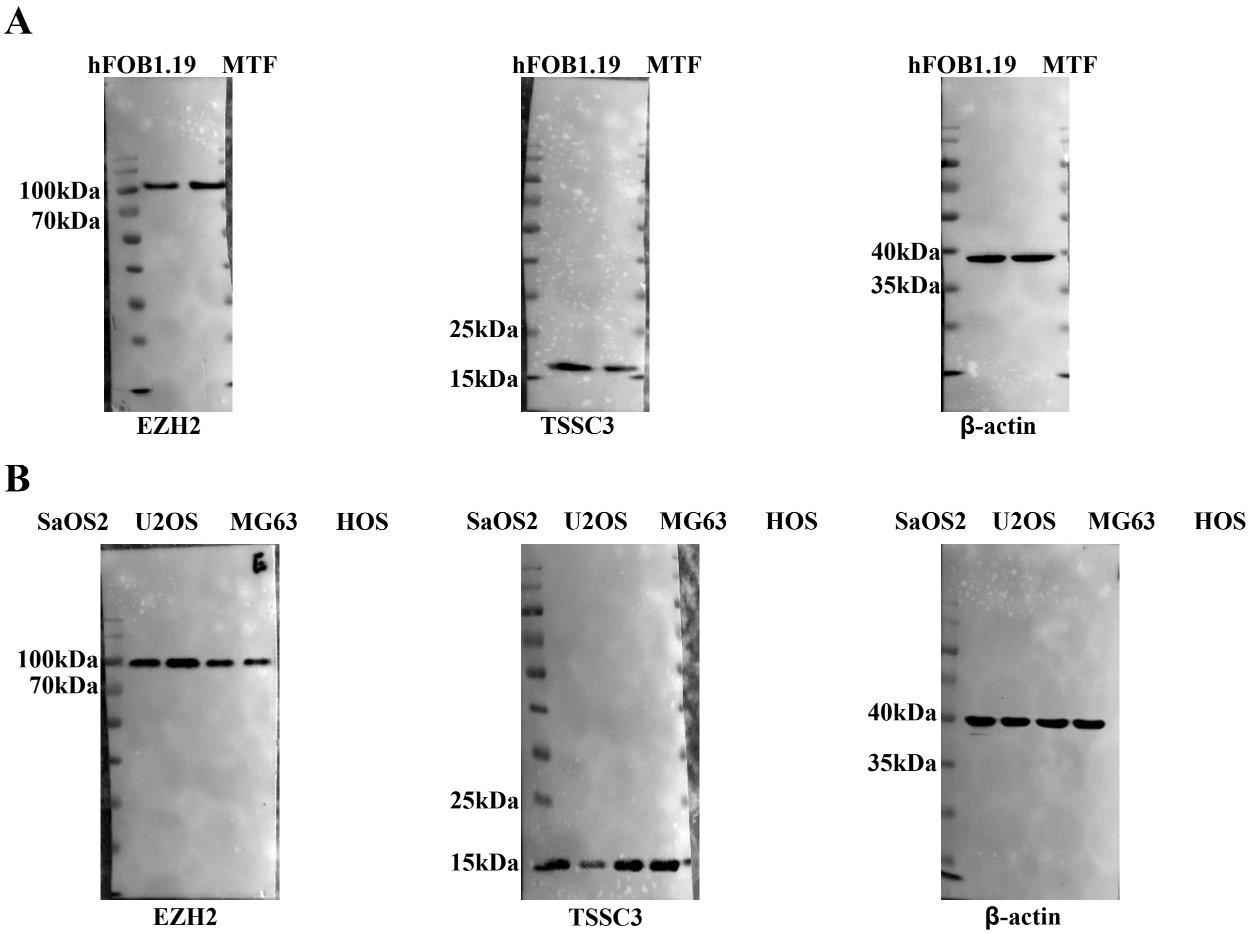

Supplementary Figure S1. (A) EZH2 is overexpressed in human osteosarcoma cell lines. EZH2 expression was significantly increased in MTF cells compared with hFOB1.19 osteoblasts, whereas TSSC3 expression was notably decreased. (B) The expression of EZH2 and TSSC3 in osteosarcoma cells. β-actin is used as a loading control. These cropped blots are used in the main figure (Figure 1) and these full-length blots are included in the supplementary figure.

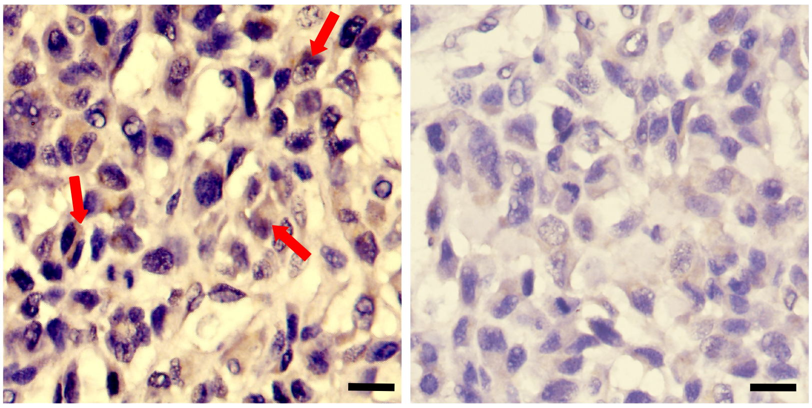

TSSC3

Supplementary Figure S2. Representative images of TSSC3 expression (200×) in osteosarcoma. The left shows positive expression (more than 50% TSSC3 positive cells) and the right shows negative expression (less than 50% positive cells) of TSSC3. The positive cells are indicated by red arrows. Scale bars: 50 μm.

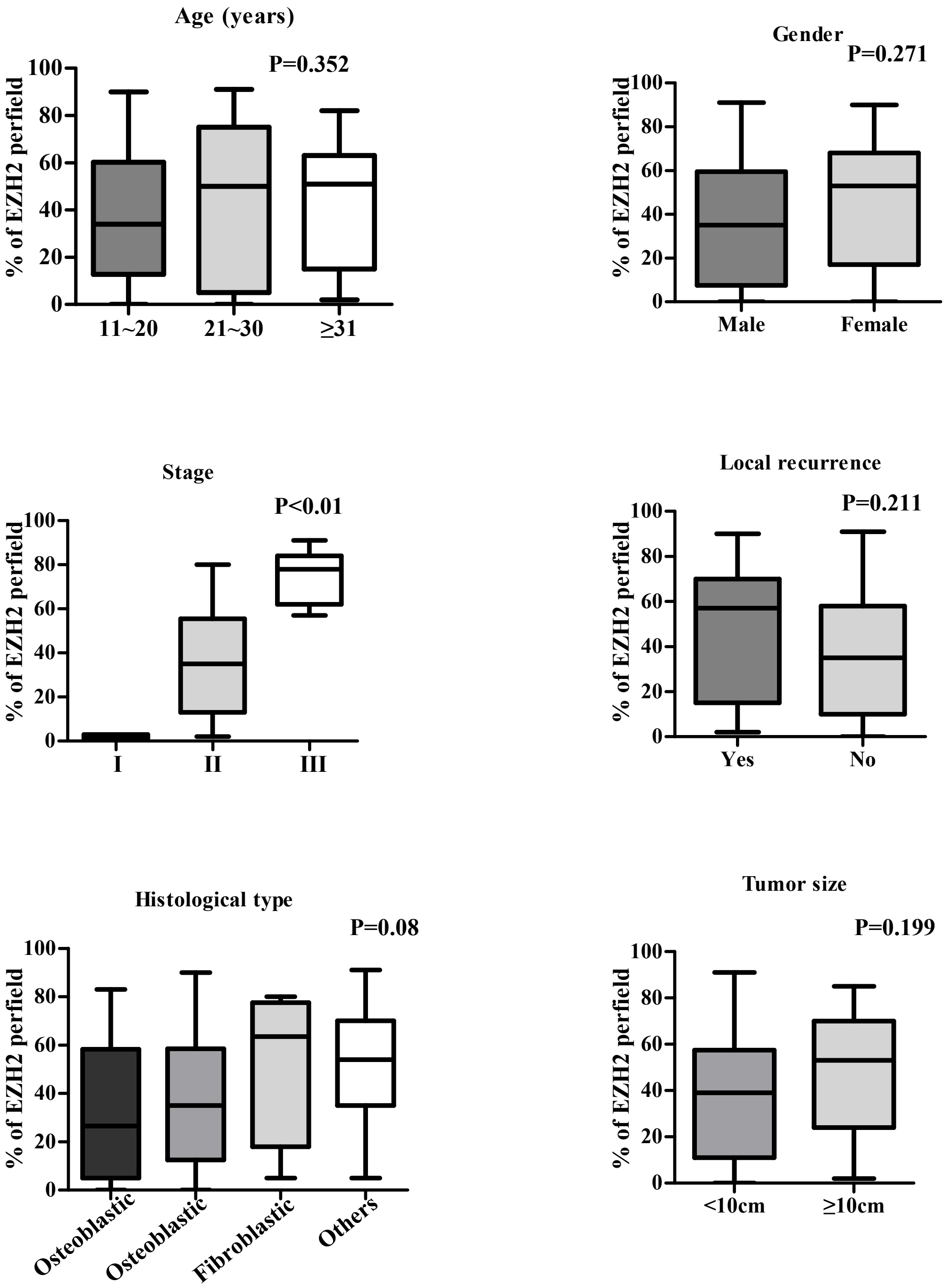

Supplementary Figure S3. Quantitative comparison of immunohistochemical staining EZH2 expression in indicated osteosarcoma subgroups.

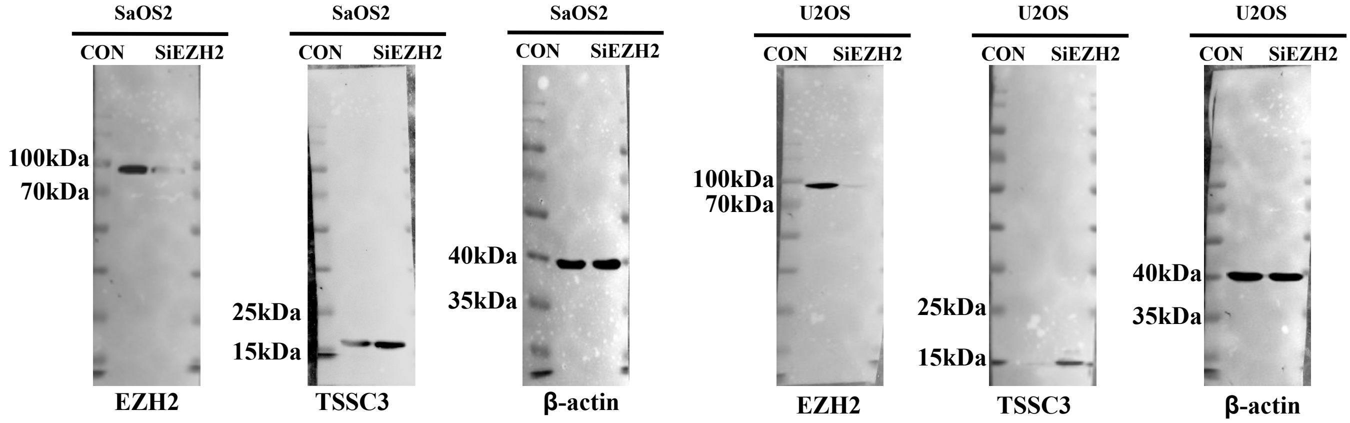

Supplementary Figure S4. Decreased protein level of EZH2 and increased TSSC3 was revealed by western blotting after transfection with lenti-shEZH2 in SaOS2 and U2OS cells. β -actin is used as a loading control. These cropped blots are used in the main figure (Figure 3) and these full-length blots are included in the supplementary figure.

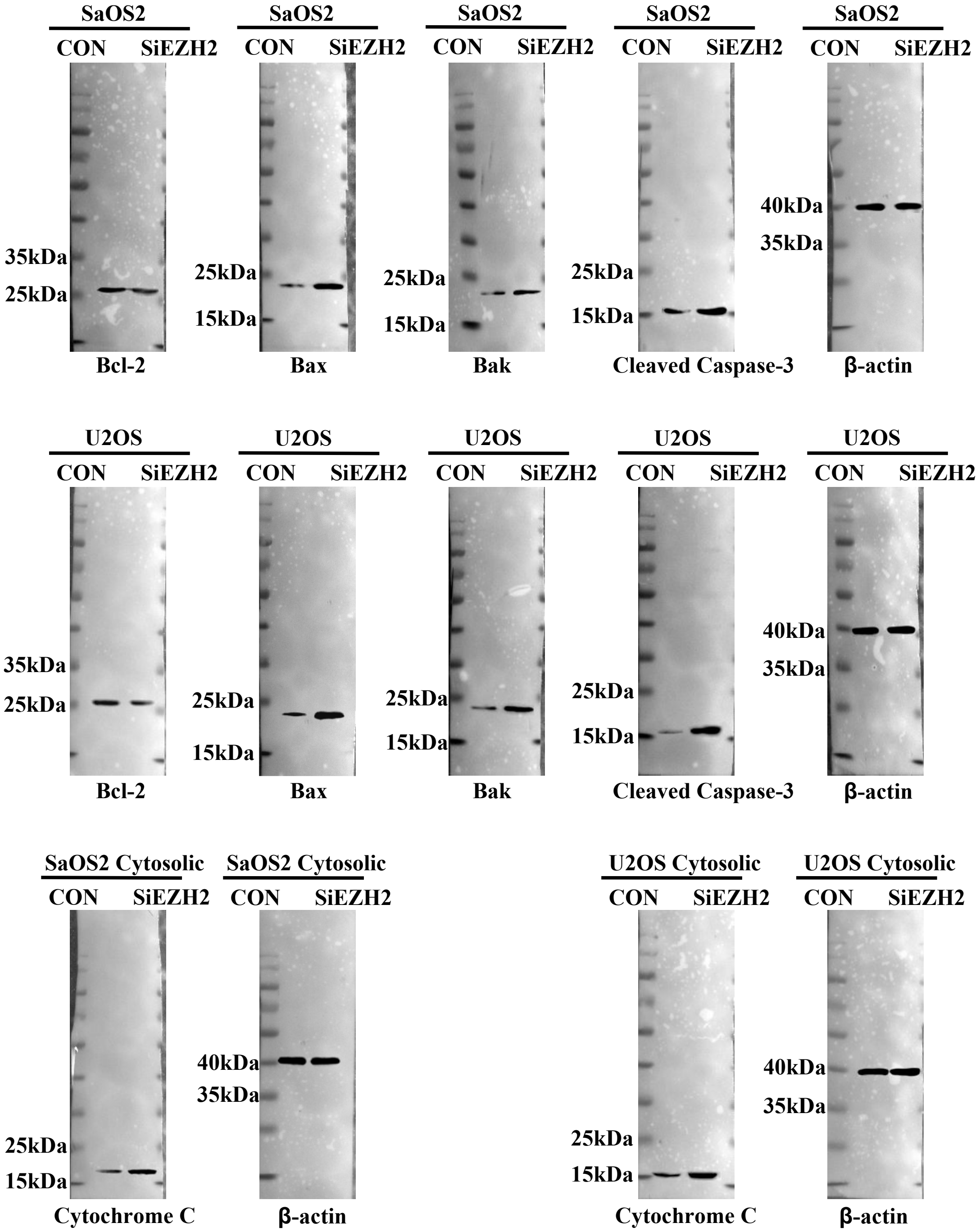

Supplementary Figure S5. Western blot analysis of Bcl-2, Bax, Bak and cleaved caspase 3 proteins in SaOS2 and U2OS cells with or without EZH2 knockdown. Western blot (lower) shows EZH2 knockdown resulted in increased expression of cytochrome C in cytosolic cell fraction. β -actin is used as a loading control. These cropped blots are used in the main figure (Figure 4) and these full-length blots are included in the supplementary figure.

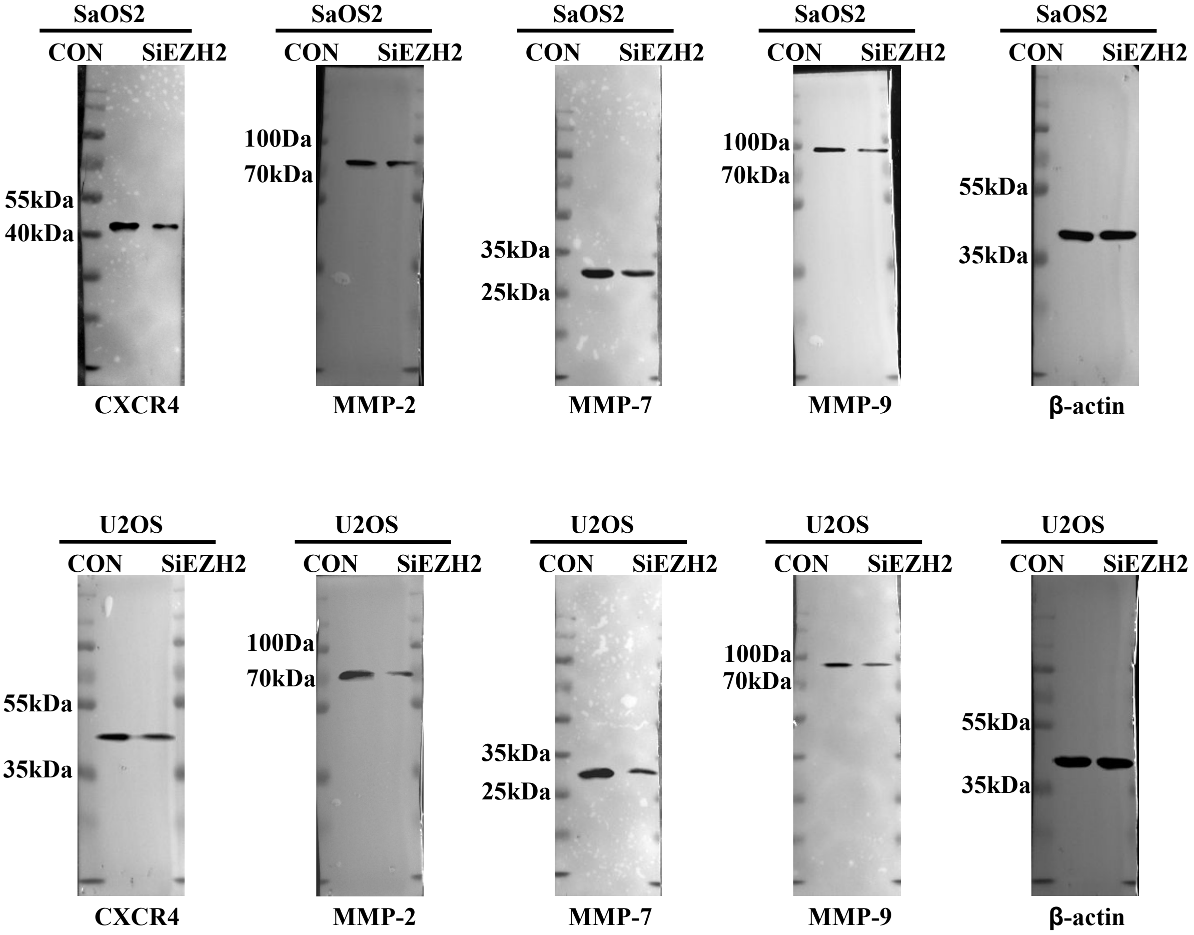

Supplementary Figure S6. Western blot analysis of CXCR4, MMP-2, MMP-7 and MMP-9 proteins in osteosarcoma cells with or without EZH2 knockdown. β -actin is used as a loading control. These cropped blots are used in the main figure (Figure 4) and these full-length blots are included in the supplementary figure.

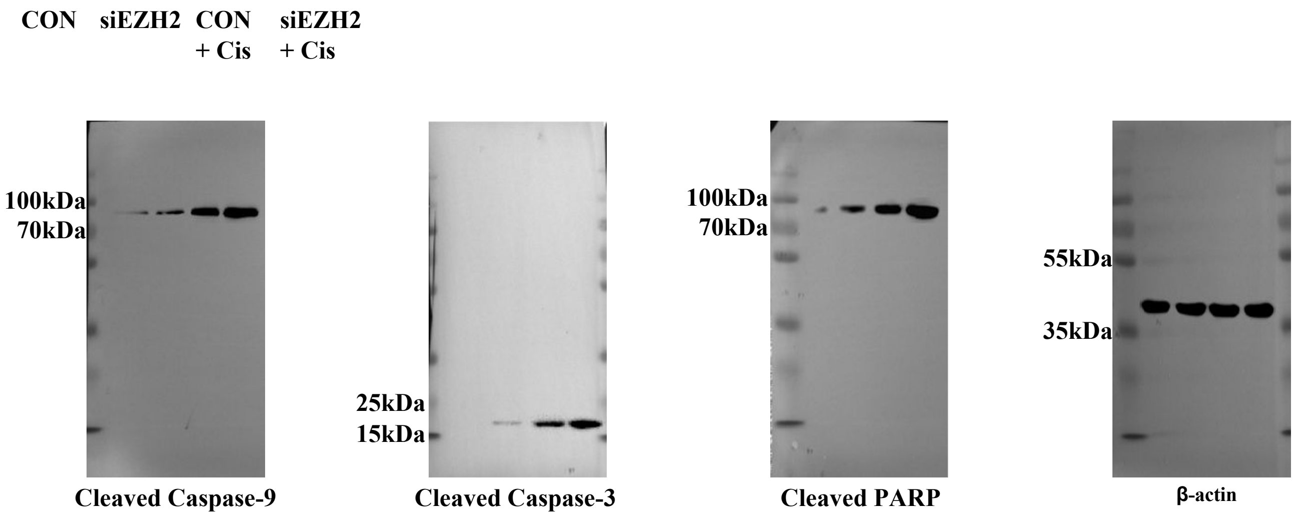

Supplementary Figure S7.Western blot analysis of cleaved caspase-9, cleaved caspase-3, and cleaved PARP proteins in osteosarcoma cells treated with or without cisplatin. β -actin is used as a loading control. These cropped blots are used in the main figure (Figure 6) and these full-length blots are included in the supplementary figure.

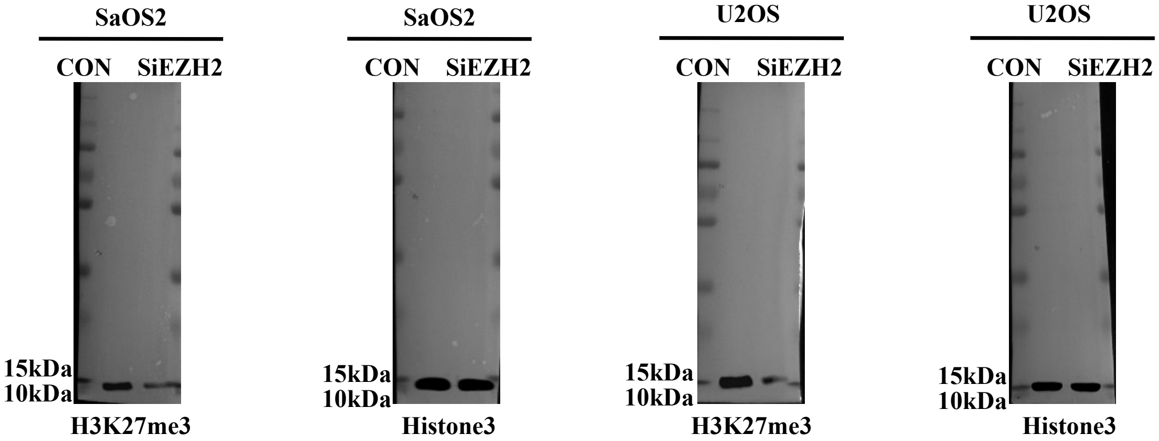

Supplementary Figure S8.Western blot analysis of HK27me3 after lenti-shEZH2 transfection using total histone 3 as a reference. These cropped blots are used in the main figure (Figure 7) and these full-length blots are included in the supplementary figure.

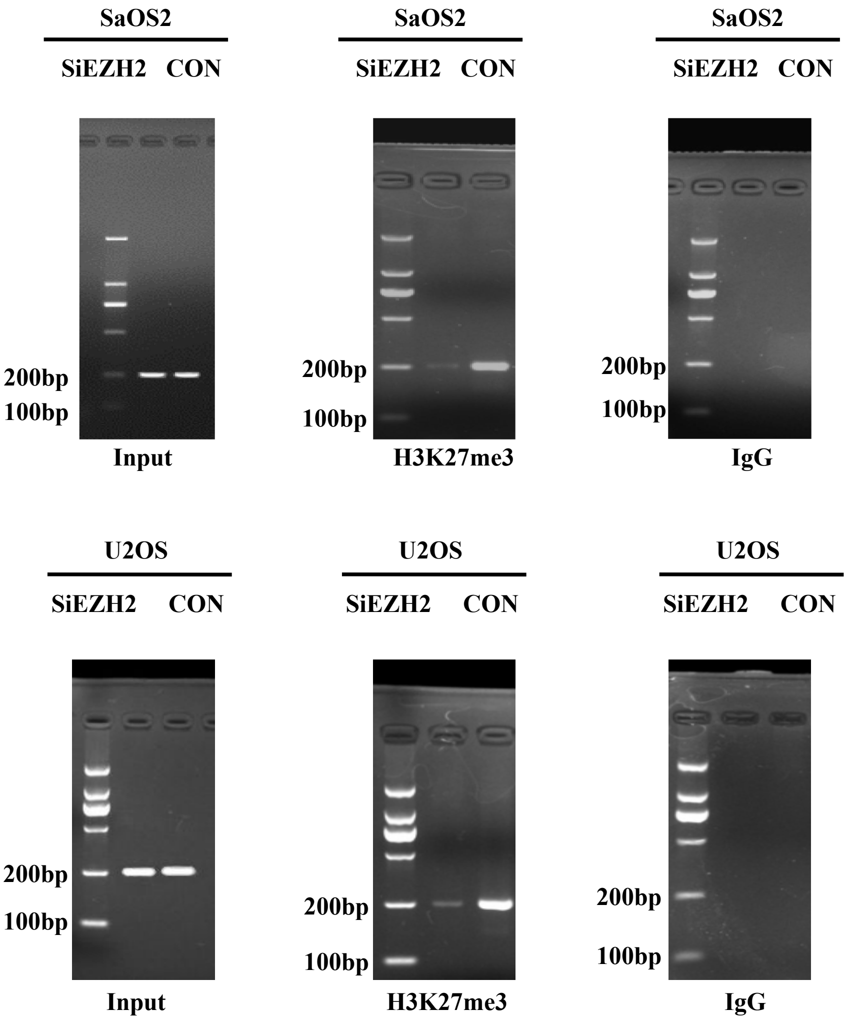

Supplementary Figure S9.ChIP analysis of H3K27me3 levels within the TSSC3 promoter, which is inhibited by EZH2 silencing. IgG was utilized as a negative control. These full-length agarose gels are supplementary for figure 7B.

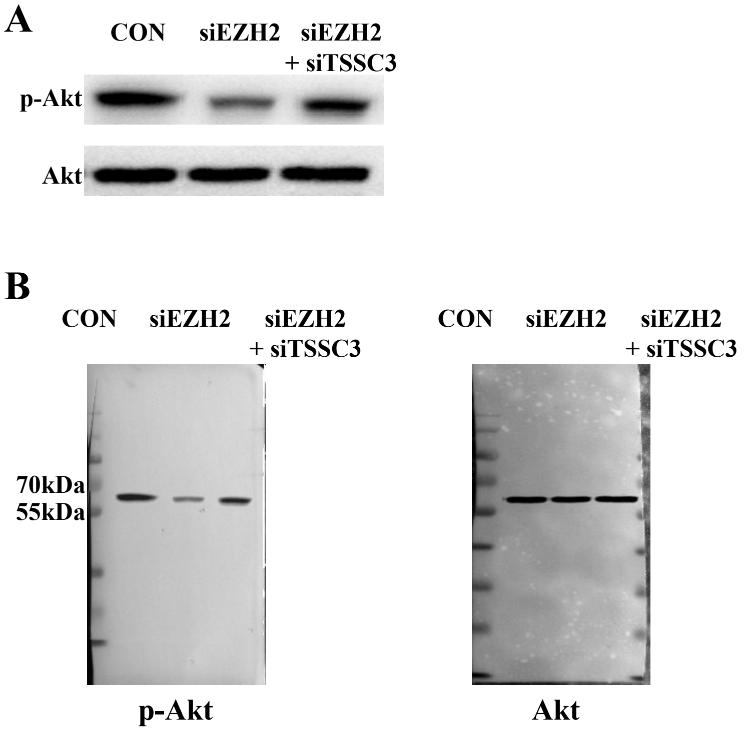

Supplementary Figure S10. (A) Western blot analysis of Akt and phosphorylated Akt in incicated cells. (B)Full-length blots of A.

Supplementary Table 1. Correlation of EZH2 and TSSC3 in human osteosarcoma tissues.

**EZH2 expression**	**TSSC3 expression**	
	**Positive**	**Negative**
Positive	7	61
Negative	11	1
P value	<0.01	
r value	-0.776	
